# Supplementary material for: Contribution from cross-country skiing, start time and shooting components to the overall and isolated biathlon pursuit race performance
Source: PLoS One. 2020 Sep 14;15(9):e0239057. doi: 10.1371/journal.pone.0239057 (PMC7489554; doi:10.1371/journal.pone.0239057)
Supplement: Attachment — Submitted filename: review.pdf [file pone.0239057.s002.pdf]

Review report on
'Contribution from cross-county skiing, start time and shooting components to the overall and isolated biathlon pursuit race performance' by Øyvind Sandbakk

March 4, 2020

1 Summary

The paper proposes an approach to evaluate the impact of several explanatory variables, like start and penalty time, on overall and isolated biathlon pursuit performance. The authors use stepwise linear regressions to identify the most important predictors. Furthermore, they use correlation analyses to examine the linear relationships between start time (penalty time, course time, shooting time, range time) and overall (isolated) pursuit race time behind. Particularly, they focus also on the differences between male and female biathletes. Additionally, they describe the relationship between sprint and pursuit races.

In general, the paper discusses an interesting topic but it partially lacks of correct descriptions and applications of statistical methods. Until now, the manuscript needs to be revised because there are fundamental issues which undermine the quality of the paper severely. Furthermore, the interpretation and discussion of the results should be expanded.

2 Major Comments

1. Stepwise linear regressions: The models used to analyze the importance of the independent variables totally explain the dependent variable because there is a perfect interrelation and, thus, the dependent variable can be perfectly computed with the independent variables. For this reason, it does not seem to make sense to estimate a linear model. Generally, stepwise regression is used to make a variable selection and not necessarily to measure the variable importance. The motivation why you should use stepwise regression to identify the variable's importance should be clearer.

   - If you want to compute the relative importance, you could also calculate the standardized coefficients and compare them with each other.

Another approach is to use the estimators of relative importance in linear regression based on variance decomposition according to Grömping [2007]. This approach allows a comparison between different metrics of relative importance.

- There is no discussion about the method you used in your stepwise regression analyses. Did you use forward, backward or both selection types to choose your models?
- Moreover, there are 1200 observations per sex. You look only on the top 30 athletes in each race. This results in 40 races but you show only findings for 38 or 37 races. Where are the other 2 respectively 3 races?
- In addition, in Table 2 you totally included 38 or 37 races to estimate the models but if you add up the number of races with best fits, you get only 34.
- The basis of each regression are 30 observations, which is a relatively small number. The interpretation of each regression result doesn't make sense because what is your general conclusion? What is the main variable which drives the general pursuit race performance? Why don't you estimate an overall model to get the relative importance of the independent variables? Definitely, it would be more difficult to get the real effects because, now, you have to consider different race effects, like course profile and so on but the result of this analysis would be more relevant for coaches.
- Moreover, there is no discussion about the assumption of linear regression analysis. Are they full-filled? Have you tested for heteroscedasticity or have you looked at the residuals' distribution? All in all, there are some problems in the methodical description and application. These problems should be discussed and explained in the paper to get a more reflected description of the method.

2. Outliers detection: Until now, there is no consistent structure and the analyses should be revised. All outliers are removed in the stepwise regression but not in the other statistical analyses. Thus, the structure is not consistent. The means, standard deviations, confidence intervals, and also the correlation analyses are biased by the outliers. For this reason, the outliers should be removed from all statistical analyses because there is no reason to include them. It would be better to use robust methods for your analyses. E.g., you could compute median, Spearman's rank correlation, a robust linear regression, and a Wilcoxon signed-rank test instead of t-test. Additionally, there are only one-dimensional outliers removed for each variable but it could also be possible that there are outliers in the two-dimensional space or multidimensional space. These outliers would also effect the statistical correlation or regression analyses.

3. Confidence interval computation: In Table 2 and 4, there are 95% confidence intervals estimated if there were more than 4 races that fitted the regression. This does not be useful because if there are 5 observations and a 95% confidence

interval is calculated than only 0.25% of the observations would be outside of the interval. In principle, it cannot be assumed that a normal distribution of the parameters exists if only 5 observations are available. Moreover, the parameter distribution could be random and, thus, the distribution is not robust. Therefore, the confidence intervals don't provide meaningfulness.

4. Correlation analyses: The correlation analysis explanation is not adequate. There has to be a better description, why a coefficient is included in the table and why not. This contributes to easier reading comprehension. Generally, the coefficients should be more interpreted. E.g., the overall pursuit race time behind correlates in 6 races positively and in 3 races negatively with range time. Thus, the coefficients cannot be interpreted adequately. The conclusion should be that there is no clear effect direction of range time on overall pursuit race time behind. All the other coefficients are positively correlated and that is as expected. So the question arises: What can we learn from the correlation analyses?

3 Minor comments

1. Line 79-80: range time and shooting time ⇒ There should be an explanation what both variables mean. A explanation in the method section is too late.
2. Line 87-88: Choking under pressure could be a reason for differences in shooting and range time compared to interval-start disciplines.
3. Line 109-114: Please, illustrate the descriptive statistics in a table. The reader could easily get an overview of the data and could compare the differences between women and men.
4. Line 117: What is the defined significance level? What are the results of the Shapiro-Wilk-tests? Are all variables normally distributed?
5. Line 117: Shapiro-Wilk test
6. Line 134: 2nd places instead of 2nd-places
7. Line 140-141: What is meant by number of places climbed between men and women? Compared to the sprint race or the world cup ranking?
8. Line 150-152: At this point, there is the problem that the means and also the confidence intervals could be extremely effected by the total meters to climb, maximum climb, the snow conditions, and weather conditions. A explanation for the reader would be helpful.
9. Line 150-152: There should be spaces between the mean and the confidence interval, e.g. $32:47\ (32:18, 33:16)$.
10. Line 154-156: There should be spaces between the mean and the confidence interval. In addition, place the second's symbol before the brackets (11.6 s (6.5,16.8)).

11. Line 157: (bib1)?
12. Table 1: There are no dependent and independent variables in a correlation analysis. You say nothing about causation in a correlation analysis. The variables' order does not matter in the coefficient's calculation.
13. Line 179-182 That is a really interesting fact but how did you compute your confidence intervals? At this point, you also have only 38 observations. It would be better to use the real data and look at the empirical outcomes.
14. Line 180: There should be spaces between the mean and the confidence interval.
15. Line 183: The significance level should be defined in the method's section. That is the correct statistical way.
16. Line 184: There should be spaces between the mean and the confidence interval.
17. Line 185: There should be spaces between the mean and the confidence interval. However, it would be preferably to use a Wilcoxon signed-rank test instead of a t-test.
18. Line 187: There should be spaces between the mean and the confidence interval.
19. Line 188-189: The percent sign should be placed after each number.
20. Table 2: See Table 1.
21. Line 209: The percent sign should be placed after each number.
22. Line 211: The percent sign should be placed after each number.
23. Line 222-223: Why are there differences between races? Is there a relationship between the differences and weather conditions, snow conditions?
24. Line 277-278: Strategic behavior could play a major role for this result.
25. Line 284: The percent sign should be placed after each number.
26. Orthography, grammar & style: In general the manuscript is well-written in style. However, there are some typos occasionally. Additional proofreading is required.

4 Recommendation

To sum up, the proposed manuscript has some fundamental statistical problems in the analysis and method description. Because of that I recommend a major revision of the manuscript. However, the paper has some serious deficiencies and cannot be published in its current form. For this reason, the statistical analysis has to be fixed.

References

Ulrike Grömping. Estimators of relative importance in linear regression based on variance decomposition. *The American Statistician*, 61(2):139–147, 2007. doi: 10.1198/000313007x188252.
