# Supplementary material for: Contribution from cross-country skiing, start time and shooting components to the overall and isolated biathlon pursuit race performance
Source: PLoS One. 2020 Sep 14;15(9):e0239057. doi: 10.1371/journal.pone.0239057 (PMC7489554; doi:10.1371/journal.pone.0239057)
Supplement: Attachment — Submitted filename: Response to reviewers (002).docx [file pone.0239057.s003.docx]

Reviewer #1: The authors present interesting data about how different components affect the biathlon pursuit performance. The paper is mostly clearly written, relatively easy to follow and is likely of importance for coaches and athletes in the biathlon sport. However, in overall, the results section is a bit messy, sometimes hard to follow.

Thank you for the positive comments and constructive feedback. We have addressed each specific comment and made changes in the manuscript accordingly. Please see specific answers to each comment below.

Specific comments:

Abstract
L38 - standing shooting positions.
L41 - shooting performance. This includes shooting time and accuracy. I would use the penalty time here as well as you have done in the main text.
L46 - Start time (i.e. sprint race performance)
L46 - explainED 80%.
L53 - pursuit race PERFORMANCE.
Yes, all comments have been taken into consideration and changes to the manuscript are done at L38,L41,L46 and L53. All changes are marked in red in the revised manuscript.

Introduction
Mostly clearly written and logical. Comments:
L59 - prone and standing shooting positions
L59-62 - Yes, biathlon is an Olympic winter sport but here it is a little bit confusing as e.g. single-mixed relay is not included in the Olympic program. Revise this sentence.
L63 - I would add Dzhilkibaeva et al. 2018 here as well.

We agree that this paper is relevant for our study and the paper is included in the manuscript accordingly. However, we argue that it does not fit after a line describing the different events in Biathlon. Dzhilkibaeva et al. (2018) investigates how results from the IBU-cup sprint races can predict performance in the World-cup sprint races and especially how course time varies between these cups.

L79 - 20% longer lap distance between shots?? Do yo mean shootings? Or Shooting stations?

Yes, this should be “shootings”. Thank you for noticing.
L93 - In addition, good shooting and... what is meant by good shooting? Fast, clear or both?

Good point. We changed this to “clean” shooting and a fast range and shooting time.

L99 - just a curiosity - how could organizers benefit from the results of this study?

In Biathlon, the international biathlon federation (IBU) is the “owner” and thus the organizer of biathlon events. Based in the findings in our investigation, the IBU can take informed decisions i.e. when designing new event types and when deciding how often a pursuit race should be arranged within a World Cup year. We have now revised this to “The International Biathlon” federation to make it clearer.

L100-105 - You should define the shooting time and range time here (or in the Introduction somewhere).
Yes we added the definition of shooting time and range time to the introduction at first mention and since course time and penalty time is already defined in the introduction we removed this information from the methods section.

Methods
L107-114 - Add information which seasons were included in the analysis (this information is in the abstract)

Yes, this has now been done.

L123 - range time excluding shooting time. This is sensitive for the outcome of your study. As shooting time is taken manually, there is a source of error. Let say, a true shooting lasts 25 seconds, +/- 2 seconds error in manual measurement means almost 10% error. When you also extract this value from the range time which is "correctly" measured using the timing-chip system, you will get a double error (error in range time as well). I understand this approach from regression analysis point of view, but you must at least mention this complex of problems in the discussion.

Thank you for your comment. Yes, shooting time is extracted from range time based on the recordings of shooting time that is also provided in the official IBU race reports to coaches and athletes. We agree with the reviewer that manual recording of shooting time is not highly accurate. However, when analyzing 37 and 38 events with 30 athletes in each race for both sexes, the amount of shootings analyzed make the statistical effect of the inaccuracy of 1-2 seconds, which goes both ways around the mean, diminishing. In addition, the shooting time has been measured in the same way for all athletes. Therefore, we would like to keep the shooting times as we have included them now, yet we have now mentioned this as a limitation in a methodological considerations paragraph.

Results
Interesting you point out that basically you must start within top 5, at least top 10, to have a chance to win the pursuit race. Good! However, consider revising the text in the results section. It is sometimes difficult to follow and one must read the same sentence a couple of times to understand what do you mean.

Thank you for this constructive feedback. We have now made changes to the results section and hope the reviewer agrees that these changes provide better readability.

L157 - remove (bib1)

We agree. This is now removed.
L164 - women (F) - should be women (W) (also in the figure 1)

Yes, this has now been changed, both in the text and in the figure.
L165 - Pearson correlation; add 'analysis'
We agree. This has been added.

Discussion
L211 - maybe add "in both sexes"

We agree. This has been added.
L227-230 - add 'in sprint'
We agree. This has been added.

L233-L240 - maybe add something about pacing & drafting here.

Yes, please see our additions on L249 and L253-256 in the revised manuscript
L243 - frequency of shootings? frequency of shooting tasks?

Yes, we changed this to frequency of shootings.

L252-253 - maybe revise to "...(5), individual (3), and according to the present results, in pursuit races.'
This has now been performed.

L273 - 60-70% in both sexes?

We agree. This has been revised.
L279 - bigger - change to larger.

We agree. This has been revised.

L287-288 - Furthermore, penalty time... of the variance in the isolated pursuit race result. You have not shown data for this conclusion. You have presented that shooting 3 and 4 explained 70-90% of the variance in total penalty time (L177).

As written in the results section and shown in table 5, our analyses show that penalty time is the most important component for the isolated pursuit race performance in almost all races among men and in around 80% of races among women, explaining approximately 62 and 54% of the variance in race time in men and women, respectively. In addition, we showed that most of the penalty time was explained by the two last shootings. We have now revised this sentence in the discussion for better readability and hope this suffices the reviewer.

Reviewer #2: The paper proposes an approach to evaluate the impact of several explanatory variables, like start and penalty time, on overall and isolated biathlon pursuit performance. The authors use stepwise linear regressions to identify the most important predictors. Furthermore, they use correlation analyses to examine the linear relationships between start time (penalty time, course time, shooting time, range time) and overall (isolated) pursuit race time behind. Particularly, they focus also on the differences between male and female biathletes. Additionally, they describe the relationship between sprint and pursuit races.

In general, the paper discusses an interesting topic but it partially lacks of correct descriptions and applications of statistical methods. Until now, the manuscript needs to be revised because there are fundamental issues which undermine the quality of the paper severely. Furthermore, the interpretation and discussion of the results should be expanded.

I recommend a major revision of the manuscript.

Thank you for taking your time to revise this manuscript accurately, and for your thorough and constructive feedback on our work. We agree with the reviewer that our methodological approach must be communicated better, and that parts of our analyses can be done differently. However, we do not fully agree that there are fundamental issues which undermine the quality of the paper severely. Please see our responses to these issues below. If there are misunderstandings or perspectives we have not seen, please challenge us further and we will do our best to revise accordingly.

When applying the same method as the below listed papers, instead of the stepwise regressions, we get approximately the same overall results as in this paper and the same ranking of the variables. See figure below using the method from these previous papers. Although the current method of pooling the data from 38 and 37 races provides some additional information on variations in the impact of various variables between races, the overall findings show that the more common mechanisms between independent variables remains the same.

Luchsinger H, Kocbach J, Ettema G, Sandbakk O. The Contribution From Cross-Country Skiing and Shooting Variables on Performance-Level and Sex Differences in Biathlon World Cup Individual Races. Int J Sports Physiol Perform. 2019;14(2):190-5. Epub 2018/07/25. doi: 10.1123/ijspp.2018-0134. PubMed PMID: 30039989

Luchsinger H, Kocbach J, Ettema G, Sandbakk O. Comparison of the Effects of Performance Level and Sex on Sprint Performance in the Biathlon World Cup. Int J Sports Physiol Perform. 2018;13(3):360-6. Epub 2017/08/05. doi: 10.1123/ijspp.2017-0112. PubMed PMID: 28771061

Major comments

1. Stepwise linear regressions: The models used to analyze the importance of the independent variables totally explain the dependent variable because there is a perfect interrelation and, thus, the dependent variable can be perfectly computed with the independent variables. For this reason, it does not seem to make sense to estimate a linear model. Generally, stepwise regression is used to make a variable selection and not necessarily to measure the variable importance. The motivation why you should use stepwise regression to identify the variable's importance should be clearer.

Thank you for your comment. We are aware that all the independent variables explain 100% of the dependent variable in our case. In our study this was done by purpose and have now been more carefully interpreted. Still, the ranking of the different independent variables in each of these 38 and 37 races are in line with the findings from the correlational analyses, the descriptive analyses and based on our common knowledge of biathlon, so the main conclusions remains the same independent of method. Altogether, both the descriptive analyses (the frequency analyses, the simple summation of start number and final result etc.), the results from the correlational analyses and the stepwise method are used to conclude in this paper. As you see below, we now additionally included and compared the standardized coefficients. This ensures a robust interpretation of the interplay between the different independent variables (race components) in biathlon pursuit races.

If you want to compute the relative importance, you could also calculate the standardized coefficients and compare them with each other.

Yes, this is now done and discussed more thoroughly in the revised manuscript.

Another approach is to use the estimators of relative importance in linear regression based on variance decomposition according to Grömping [2007]. This approach allows a comparison between different metrics of relative importance.

Thank you for the suggestion. We have considered this method but chose to stick to our approach in addition to adding standardized coefficients in the revised manuscript.

There is no discussion about the method you used in your stepwise regression analyses. Did you use forward, backward or both selection types to choose your models?

In the case of stepwise regression, we directly included all variables in the regression (that in total explain 100% of the variance), and the model defines the ranking of variables.

Moreover, there are 1200 observations per sex. You look only on the top 30 athletes in each race. This results in 40 races but you show only findings for 38 or 37 races. Where are the other 2 respectively 3 races?

It is unclear how the reviewer concludes that there are 1200 observations. As described in the methods section there are 1140 results among men and 1110 results among women, originally. 1140/30 = 38 races among men and 1110/30 = 37 races in women.

In addition, in Table 2 you totally included 38 or 37 races to estimate the models but if you add up the number of races with best fits, you get only 34.

Yes, this is correct. There are 4 and 3 races among men and women not included in the original table 2 (now table 3). In addition, for the isolated pursuit race performance analysis shown in table 5 in the revised manuscript, two races among women were not included. These races were not included because in each race the rankings of the variables varied in both sexes. Including these special races would create an unnecessary long table since the most important findings were covered by model outcome 1 and 2 in both the overall and isolated pursuit race performance analyses. However, we agree with the reviewer that these results should also be included somehow, in order for the reader to consider all findings in their interpretation of the results.

We have now added a sentence after table 2: “In addition to the results in table 2, three races in men and two races in women fit 3 additional models with various rankings of the different variables and in one race among men no variables correlated with overall performance.”

Accordingly, we added the following sentence after table 5: “In addition to the results in table 5, two races among women fit two different models with different rankings of the variables.”

The basis of each regression are 30 observations, which is a relatively small number. The interpretation of each regression result doesn't make sense because what is your general conclusion? What is the main variable which drives the general pursuit race performance? Why don't you estimate an overall model to get the relative importance of the independent variables? Definitely, it would be more difficult to get the real effects because, now, you have to consider different race effects, like course profile and so on but the result of this analysis would be more relevant for coaches.

Thank you for your constructive criticism of this part of the methodology. Top 30 athletes were chosen to ensure that athletes were motivated to “do their best” as they are still competing for World Cup points. Athletes between 30^th^ and 60^th^ place are likely to be less motivated especially in the end of a race and this would affect the results.

Although an overall result grouping all the races into one model would provide and overall view of the importance of the race factors, we disagree with the reviewer that this would provide more valuable information for coaches. As the results are provided now, the reader can evaluate if 23/38 races with start time being the most important factor among men is many or few races. If a more general result was to be provided, the results in model outcome 2 would affect the results in model outcome 1 and vice-versa. Thus, a more unclear interpretation of the results would be provided, in our opinion. We would off course reconsider this if the reviewer provides us with new perspectives that we have not yet seen.

Moreover, there is no discussion about the assumption of linear regression analysis. Are they full-filled? Have you tested for heteroscedasticity or have you looked at the residuals' distribution? All in all, there are some problems in the methodical description and application. These problems should be discussed and explained in the paper to get a more reflected description of the method.

We agree with the reviewer that a better explanation of the choices for methodology should be provided. Stepwise multiple regression analysis has been used with success in several papers ranking the different components to the overall performance were the number of participants was lower than 30. We looked at the residual plots and concluded that with the removal of outliers, the assumptions for using linear regression were met. This is added to the revise manuscript.

2. Outliers detection: Until now, there is no consistent structure and the analyses should be revised. All outliers are removed in the stepwise regression but not in the other statistical analyses. Thus, the structure is not consistent. The means, standard deviations, confidence intervals, and also the correlation analyses are biased by the outliers. For this reason, the outliers should be removed from all statistical analyses because there is no reason to include them. It would be better to use robust methods for your analyses. E.g., you could compute median, Spearman's rank correlation, a robust linear regression, and a Wilcoxon signed-rank test instead of t-test. Additionally, there are only one-dimensional outliers removed for each variable but it could also be possible that there are outliers in the two-dimensional space or multidimensional space. These outliers would also effect the statistical correlation or regression

analyses.

For the stepwise and the correlation analyses, as well as for the descriptive analyses related to these, the outliers were removed. It was just a mistake when it was written in the methods section that outliers were included in the correlation analyses and all the descriptive analyses. We apologize for this! Thank you for noticing. It would make little sense to do this as the stepwise method and correlation analyses are performed at the same time in SPSS. We agree with the reviewer, however, that there is an inconsistency in the inclusion and exclusion of outliers for the rest of the results. As winners and 2^nd^ places are often outliers, removing these results when performing analyzes on winners or the fastest isolated pursuit race times is not a good idea. We argue that the strength of this study is that we use different methods to observe our data and that both simple descriptive statistics, correlations and linear regression provide insights from different angles. This ultimately provides a total overview that can be used for robust conclusions. In addition, we agree that a more comprehensive discussion about the outliers and the methodology should be included.

3. Confidence interval computation: In Table 2 and 4, there are 95% confidence intervals estimated if there were more than 4 races that fitted the regression. This does not be useful because if there are 5 observations and a 95% confidence interval is calculated than only 0.25% of the observations would be outside of the interval. In principle, it cannot be assumed that a normal distribution of the parameters exists if only 5 observations are available. Moreover, the parameter distribution could be random and, thus, the distribution is not robust. Therefore, the confidence intervals don't provide meaningfulness.

In our opinion, we do not follow this reasoning. In “Fundamentals of biostatistics”, Rossner (2008 11 ed) describes how meaningful confidence intervals with only 5 observations are generally provided. However, if there are less than 5 observations confidence intervals using standard methods should be interpreted with care. We agree with the reviewer and there is no doubt that CI from groups with a low n has little of the dataset outside the interval. The interval itself, however, is still meaningful and provides a probability that the true mean lies within this interval.

4. Correlation analyses: The correlation analysis explanation is not adequate. There has to be a better description, why a coefficient is included in the table and why not. This contributes to easier reading comprehension. Generally, the coefficients should be more interpreted. E.g., the overall pursuit race time behind correlates in 6 races positively and in 3 races negatively with range time. Thus, the coefficients cannot be interpreted adequately.

The conclusion should be that there is no clear effect direction of range time on overall pursuit race time behind. All the other coefficients are positively correlated and that is as expected. So the question arises: What can we learn from the correlation analyses?

We agree with the reviewer that the correlation analyses should have been described better. This is done in the revised manuscript. For clarity, only significant correlations were included in the table. Thus, in 35 races among men and 37 races among women, start time correlated significantly with overall performance. The average of the correlation coefficients for these significant cases were listed in the table.

Minor comments

1. Line 79-80: range time and shooting time. There should be an explanation what both variables mean. An explanation in the method section is too late.

Yes, this has now been added to the introduction

2. Line 87-88: Choking under pressure could be a reason for differences in shooting

and range time compared to interval-start disciplines.

Yes, the paper of Vickers and Williams from 2008 investigating the effects of physiological arousal, cognitive anxiety and gaze control in Biathlon has now been added to the introduction

3. Line 109-114: Please, illustrate the descriptive statistics in a table. The reader could easily get an overview of the data and could compare the differences between women and men.

Revised accordingly.

4. Line 117: What is the defined significance level? What are the results of the

Shapiro-Wilk-tests? Are all variables normally distributed?

All variables, except start number and the number of misses (as the athlete can’t miss less than 0 shots), were normally distributed. For start number analyses we now used the median values but for the number of misses we kept mean and 95% CI since mean +- CI provide more insight to the shooting performance than just the median misses in both sexes and we argue that there is enough data in each race to neglect the minor skewness in this variable.

Normal curve on bar plots for frequency analysis of Independent variables for all races, among men:

Normal curve on bar plots for frequency analysis of Independent variables for all races, among women:

5. Line 117: Shapiro-Wilk test

Yes, this is now changed in the revised manuscript.

6. Line 134: 2nd places instead of 2nd-places

Revised accordingly.

7. Line 140-141: What is meant by number of places climbed between men and women? Compared to the sprint race or the world cup ranking?

The difference between start number and final rank in each pursuit race. We have now included that there might in some cases be a few athletes who finished top 60 in the sprint race not competing in the pursuit race, which could affect the analyses of climbed places to a minor degree.

8. Line 150-152: At this point, there is the problem that the means and also the

confidence intervals could be extremely effected by the total meters to climb,

maximum climb, the snow conditions, and weather conditions. An explanation

for the reader would be helpful.

We thank the reviewer for pointing this out. A more thorough methodological consideration chapter is added to the manuscript before the conclusions.

9. Line 150-152: There should be spaces between the mean and the confidence

interval, e.g. 32 : 47 (32 : 18; 33 : 16).

We agree to the written comment but suggest that the mean and CI are written 32:47 (32:18 ; 33:16)

10. Line 154-156: There should be spaces between the mean and the confidence interval. In addition, place the second's symbol before the brackets (11.6 s (6.5,16.8)).

This has now been added.

11. Line 157: (bib1)?

Means start number 1 but this is now removed accordingly since the sentence itself describe this well enough. Thank you for noticing this detail.

12. Table 1: There are no dependent and independent variables in a correlation

analysis. You say nothing about causation in a correlation analysis. The

variables' order does not matter in the coefficient's calculation.

13. Line 179-182 That is a really interesting fact but how did you compute your

confidence intervals? At this point, you also have only 38 observations. It

would be better to use the real data and look at the empirical outcomes.

Thank you for your comment. In total, there are 38 separate athletes (as there are 38 races) having the fastest isolated pursuit race time. The mean values with confidence intervals provide useful information to coaches and athletes because it shows that the fastest isolated race times are mostly (CI95%) gained by people starting from position 16-25 in men and 10-17 in women. The point of providing descriptive statistics is to avoid presenting the whole dataset with individual outcomes. Therefore, we do not understand what is meant by “use real data” as the descriptive results presented here reflect the data. However, we understand that it is somewhat confusing mixing the results from the correlation analyses, linear regression and the simple descriptive statistics and have tried to write the results section more clearly.

It should also be noted that in a previous comment by the reviewer it was suggested that there should be more consistency in including and removing outliers. If we were consistent in removing outliers also from the descriptive results (which do affect linear regression analyses to a greater degree than these mean values) this exact result would be lost. Therefore, we argue that this emphasizes the importance of looking at this special dataset with different methods.

14. Line 180: There should be spaces between the mean and the confidence interval.

Revised accordingly.

15. Line 183: The significance level should be defined in the method's section.

That is the correct statistical way.

Changed accordingly.

16. Line 184: There should be spaces between the mean and the confidence interval.

Changed accordingly

17. Line 185: There should be spaces between the mean and the confidence interval.

Changed accordingly

However, it would be preferably to use a Wilcoxon signed-rank test

instead of a t-test.

All variables except number of climbed places were normally distributed, and we also checked if our analyses differed between t-test and Wilcoxon signed-rank; this was not the case in any of the cases examined. After discussion with a statistician we therefore chose to stick to t-tests.

18. Line 187: There should be spaces between the mean and the confidence interval.

Revised.

19. Line 188-189: The percent sign should be placed after each number.

Revised.

20. Table 2: See Table 1.

Revised.

21. Line 209: The percent sign should be placed after each number.

Revised.

22. Line 211: The percent sign should be placed after each number.

Revised.

23. Line 222-223: Why are there di_erences between races? Is there a relationship

between the di_erences and weather conditions, snow conditions?

This would be interesting to know more about. However, it is outside the scope of this study. In addition, the weather measurements by IBU that is reported in the official race reports provide an overall impression of the weather conditions that day but are too little sensitive to be useful in such an analysis as this. The measurements are carried out 30 minutes before the start, at the start time and 30 minutes after the race but to be useful in this case one should know the ski-snow friction for the first athletes vs. the athletes chasing from behind etc.

24. Line 277-278: Strategic behavior could play a major role for this result.

Yes, we agree. This is now further clarified in the revised manuscript.

25. Line 284: The percent sign should be placed after each number.

Revised.

26. Orthography, grammar & style: In general the manuscript is well-written in

style. However, there are some typos occasionally. Additional proofreading is

required.

Thank you for noticing. We have now revised the manuscript and tried to clean it for typos accordingly.

4 Recommendation

To sum up, the proposed manuscript has some fundamental statistical problems in

the analysis and method description. Because of that I recommend a major revision

of the manuscript. However, the paper has some serious deficiencies and cannot be

published in its current form. For this reason, the statistical analysis has to be fixed.

Reviewer #3: The article submitted for review covers important and current issues of determinants of sport results. Structure of the work is correct. It includes original research problem, which is characterized properly and transparently. Statistical methods chosen well. The work is written well.

Thank you for this positive comment and the recommendation of our work.
