# Supplementary material for: Contribution from cross-country skiing, start time and shooting components to the overall and isolated biathlon pursuit race performance
Source: PLoS One. 2020 Sep 14;15(9):e0239057. doi: 10.1371/journal.pone.0239057 (PMC7489554; doi:10.1371/journal.pone.0239057)
Supplement: Attachment — Submitted filename: Response to reviewers revision 2 Luchsinger.docx [file pone.0239057.s004.docx]

Revision 2

Reviewer #1: I'm happy with the revision the authors have made. I have only one minor issue:

L343: "Extracting the shooting time from Range time" You propose that the error marginal is 1 sec but when thinking of pursuit competition and within the races you have been studying, there is definitively several cases when there are quite a many biathletes approaching and/or leaving the shooting line at the same time. This means that it is impossible for the volunteers to push "several buttons" at the same time. I agree that the error is random, but I suggest to revise the last sentence in methodological considerations. Something with style: "However, the error is random but this possible error needs to be considered when interpreting the of the present study."

Thank you for your advice upon this subject. We have now adjusted the last sentence in the methodological considerations paragraph according to the suggestion provided. Please see changes marked in yellow in the revised manuscript. We kept the previous changes in red color.

Reviewer #2: First of all, I want to thank the authors for the detailed answers to the comments of the first review. The authors have revised their manuscript precisely. Nonetheless, there is a major limitation of the analysis and it would be appreciated if the authors would add the limitation in their manuscript for publication. The added methodological considerations section is a first step to pick up the limitations of the analysis. But I think one major limitation, which was also mentioned in the first review, should be clearly communicated to the reader. As for each race, an individual regression model is fitted, the results are only valid for this race. Thus, no generalization on the basis of the regression results holds, which means that the results should be interpreted conservatively. Your answer in the first review is correct: ''As the results are provided now, the reader can evaluate if 23/38 races with start time being the most important factor among men is many or few races. If a more general result was to be provided, the results in model outcome 2 would affect the results in model outcome 1 and vice-versa.". For this reason, I think an additional paragraph should be added to the methodological considerations section that clarifies the limitation and ensures that the reader doesn't make wrong conclusions.

Additionally, the independent variables are likely to have high correlations (although no multicollinearity statistics are provided), which could bias the results. Thus, it would not clear which variable contributes to the dependent variable. This could invalidate the statements on the importance of certain variables. For this reason, it is necessary to check if this problem arises. Should this be the case then the authors have to find a solution to this problem because as mentioned above it would lead to wrong conclusions. Otherwise, they should emphasize that no problem on the basis of correlation exists.

To sum up, I appreciate the changes in the manuscript. The authors should clearly mention the problem of generalization in their manuscript and they should check if the problem of high correlations between the independent variables exists. For this reason, I recommend a minor revision if the problem of high correlations does not occur.

Thank you for your thorough reply and positive criticism of this manuscript. We have now added additional information about the implications of analyzing each race individually in the methodological considerations section in the revised manuscript. Please see changes marked in yellow in the revised manuscript. We kept the previous changes marked in red color.

We found significant multicollinearity between a few independent variables in some of the races, but the correlation coefficients of these associations were relatively low (mostly 0.3-0.4 and never above 0.6). Although the results of the linear regression analyses must be interpreted with this in mind, we argue that this multicollinearity between independent variables did not affect the conclusions of our study. This is supported by the consistent findings across the various analyses done in our approach. We added a paragraph about this in the methodological consideration section.
